# Use of drama for improving breastfeeding initiation, exclusive breastfeeding and breastfeeding self-efficacy among rural pregnant women from selected communities in two Local Government Areas (LGAs) in Ibadan, Nigeria

**DOI:** 10.1371/journal.pone.0290130

**Published:** 2024-08-29

**Authors:** Yetunde Omotola Ogundairo, Adepoju Oladejo Thomas, Olumide Adesola Olufunmilola

**Affiliations:** 1 Department of Human Nutrition and Dietetics, Faculty of Public Health, College of Medicine, University of Ibadan, Ibadan, Oyo State, Nigeria; 2 Institute of Child Health, Faculty of Clinical Sciences, College of Medicine, University of Ibadan and University College Hospital, Ibadan, Oyo State, Nigeria; Ogun State College, College of Nursing, NIGERIA

## Abstract

**Background:**

Breastfeeding self-efficacy (BFSE) is a key variable that enhances exclusive breastfeeding (EBF) and promotes positive health outcomes for infants and their mothers. To increase BFSE and EBF of mothers, numerous interventions targeting prenatal and postnatal periods have been developed. However, there is paucity of studies utilizing drama interventions for improving BFSE and EBF.

**Objectives:**

This study assessed the effect of a drama intervention on BFSE, initiation, and EBF of pregnant women in rural communities in Lagelu and Egbeda Local Government Areas (LGAs), Ibadan, Oyo State, Nigeria.

**Methodology:**

A quasi-experimental study was conducted with pregnant women in their second trimester. Selected communities from Lagelu and Egbeda LGAs were randomized into experimental and control groups. A total of 200 pregnant women (100 experimental and 100 control groups) were enlisted and followed-up at one, three and six months postnatal periods. A six-session programme comprising four episodes of drama and two sessions on hygiene practices were presented to experimental groups prior to delivery, while the control group received health talk on hygiene practices. Computer-Assisted Personal Interview (ODK) was used to obtain information on socio-demographic, BFSE, initiation, and EBF at prenatal and postnatal periods. BSFE scores were categorized as low (14–32), average (33–51), and high (52–70). Descriptive and inferential statistics was used to analyze data at α0.05.

**Results:**

Mean ages of women were 28.4 ±6.5 and 27.0±6.2years in experimental and control groups respectively. Average and high BFSE pre-intervention (11.0%; 89.0% and 9.0%; 91.0%) and six months post-intervention (97.3%;100% and 95.2%; 95%) for experimental and control groups. Age, marital status, and occupation were predictors of BFSE, breastfeeding initiation and EBF at (R^2^ = 22.3; p<0.05).

**Conclusion:**

The experimental group had an increase in BFSE, initiation, and EBF practice compared to control group. The use of drama intervention is recommended for effective breastfeeding practices.

## Introduction

Breastfeeding self-efficacy (BFSE) enhances a mother’s confident ability to breastfeed her infant without considering the challenges involved. It predicts a mother’s decision about breastfeeding, her preferred method of feeding her infant, the kind of effort she plans to invest in this process, her thought pattern, as well as her emotional reactions to breastfeeding difficulties [1]. Several studies have identified the linkage between BFSE, breastfeeding intention, initiation, duration, and exclusivity [2, 3]. These studies have employed four drivers of BFSE to increase the confidence of mothers in breastfeeding their infants [4]. Besides, during prenatal and postnatal periods, different educational materials such as flip charts, pamphlets, videotapes, skin-to-skin contact and health talks are used to help mothers increase their BFSE. Findings from these measures have revealed a positive increase in BFSE as well as breastfeeding outcomes at four weeks, eight weeks and twelve weeks postpartum respectively [5–7].

Drama which has a performance and interdisciplinary attribute for multiple representations and understanding of important questions as well as ensuring the synergy between cognitive and emotional domains, has been used in the health sector. However, little is known about the use of drama intervention to improve BFSE, breastfeeding initiation, and EBF practices of mothers. Van de Water (2021:3) noted that research from other disciplines such as sociology, neuroscience, and psychology are beginning to embrace the position of drama practitioners that “human instincts call for embodied and contextualized learning”. Drama not only encourages participants and viewers to envision possibilities in their thoughts but also creates opportunities for them to express abstractly conceived ideas [8]. Acknowledging the death of information on the use of drama for improving mothers’ breastfeeding practices, this study was therefore designed to assess the use of drama intervention to improve BFSE, breastfeeding initiation, and EBF practices among women from selected communities in Ibadan, Oyo State, Nigeria.

## Methodology

### Study design and participants

A quasi-experimental study was conducted for a period of 18 months (June 2020 to December 2021). A total of 200 pregnant women in their second trimester who gave their voluntary consent were recruited from selected rural communities in Egbeda and Lagelu LGAs and randomized into the experimental (n = 100) and control groups (n = 100).

### Study instruments

A pre-tested, interviewer administered questionnaire consisting of five sections was used to obtain information from research participants. Each of the five sections covers the following: maternal socio-demographic characteristics, pregnancy related issues (pregnancy trimesters, parity, mode of delivery, antenatal registration, and sources of care), BFSE, breastfeeding initiation, and EBF practices. BFSE was assessed with breastfeeding self-efficacy short form tools (BSES-sf) 14-item scale revised by Dennis [4]. A total of 14 questions were asked with each response graded on a five-point Likert scale: 5 = Very Confident, 4 = Confident, 3 = Sometimes confident, 2 = not very confident, and 1 = not at all confident. The total score from each respondent was categorized as low self-efficacy (14–32), average self-efficacy(33–51) and high self-efficacy (52–70) from studies in Poland [9] and Brazil [10]. Breastfeeding initiation and EBF were assessed using the breastfeeding practices questions by Agho [11].

### Data collection

A Computer-Assisted Personal Interview was used to obtain information from respondents at baseline and endline (one, three and six months postpartum). At baseline, information on socio-demographic characteristics, pregnancy-related issues, and BFSE were obtained. Also, at baseline, a breastfeeding chart containing the names, expected date of delivery, and telephone numbers of the participants and their spouses was developed to monitor each respondent delivery day for the collection of other follow-up data. Additionally, peer-support women were recruited from these selected communities to assist in monitoring the research participants in order to provide daily delivery reports on the day of their delivery. These measures facilitated prompt collection of follow-up date from research participants after delivery.

The first follow-up was conducted within one week postpartum to obtain information on their breastfeeding initiation time. At one month postpartum, the second follow-up was conducted to assess their confidence on EBF practice. At three and six months postpartum, the third and fourth follow-up were conducted to assess the mother’s level of confidence and EBF practices.

### Intervention and control dose

A drama-based intervention was implemented. The intervention incorporated the four drivers of BFSE such as personal accomplishment, verbal persuasion, vicarious experience and physiological affection to increase the breastfeeding self-efficacy of the intervention women prior to their delivery. The recorded 80-minute four-episode drama-based intervention was aired over two weeks in the open village squares. This was done concurrently in the two LGAs. During the first session, the women viewed two episodes of the drama on early initiation of breastfeeding, the importance of colostrum, proper positioning and attachments of the baby to the breast, and mother’s perception of insufficient breast milk. The second session on breast size and quantity of breastmilk, benefits of breastfeeding to mother, child and community and mothers past and present breastfeeding experiences was also viewed at the second week. At the end of each episode, there was a brief discussion with the participants to assess their level of understanding and knowledge. This was used as a basis for further understanding in the subsequent episodes.

For participants in the control group, they received a two-episode, step-by-step practical session on hand washing and personal hygiene.

### Ethical approval

The study received ethical approval (Reference no: UI/EC/19/001) from the Institute for Advanced Medical Research and Training (IAMRAT), University College Hospital and University of Ibadan Institutional Ethical Review Committee on May, 20, 2019. All the research participants provided a written informed consent prior to the commencement of the study. Participation in the study was voluntary and all the information obtained from the study were kept confidential.

### Data analysis

Baseline data were summarized with descriptive statistics. Independent t-test was used to compare mean differences in BFSE, Breastfeeding initiation and EBF practices between the two groups. Chi-square was used to test for association between BFSE and EBF practices between the two groups. Repeated measure of Analysis of Variance (ANOVA) was used to determine change in breastfeeding self-efficacy mean scores from baseline to endline in the two groups. In addition, logistic regression was used to identify other predictors of BFSE and EBF at p<0.05.

## Results

**Table 1** explains the socio-demographic characteristics of women in the study group. The overall mean ages of the respondent were 27.00±6.20 and 28.40±6.50 for intervention and control groups respectively. Slightly above average (53.5%) were between 20–29 years of age, and 52.5% were Christians. Almost all (97.0%) participants were married, 90.5% were Yoruba, and 60% had a secondary level of education as their highest formal education attainment. Most of the participants (89.5%) earned less than the government-regulated monthly minimum wage of ₦30,000.00, and 37.5% were traders. There was no significant difference in the socio-demographic characteristics of the participants in the experimental and control groups (p>0.05).

**Table 1:** Socio-demographic characteristics of participants.

Variable	Control N (%)	Intervention N (%)	Total	p-value
**Age in Years**				
<20years	7(7.0)	8(8.0)	15(7.5)	0.550
20–29years	49(49.0)	58(58.0)	107(53.5)	
30–39years	39(39.0)	30(30.0)	69(34.5)	
**Ethnicity**				
Yoruba	97(97.0)	84(84.0)	181(90.5)	0.018
Igbo	2(2.0)	3(3.0)	5(2.5)	
Hausa	0(0.0)	5(5.0)	5(2.5)	
TIV/Benue	0(0.0)	4(4.0)	4(2.0)	
Others	1(1.0)	4(4.0)	5(2.5)	
**Religion**				
Christianity	46(46.0)	59(59.0)	105(52.5)	0.066
Islam	54(54.0)	41(41.0)	95(47.5)	
**Marital status**				
Single	1(1.0)	5(5.0)	6(3.0)	
Married	99(99.0)	95(95.0)	194(97.0)	0.097
**Educational Level**				
None	4(4.0)	8(8.0)	12(6.0)	0.405
Primary	21(21.0)	27(27.0)	48(24.0)	
Secondary	65(65.0)	55(55.0)	120(60.0)	
Post-Secondary	10(10)	10(10)	20(10.0)	
**Occupation**				
Artisan	43(43.0)	30(30.0)	73(36.5)	0.049
Trader	39(39.0)	36(36.0)	75(37.5)	
Full housewife	11(11.0)	20(20.0)	31(15.5)	
Others	7(7.0)	8(8.0)	15(7.5)	
Farmer	0(0.0)	4(4.0)	4(2.0)	
Civil servants	0(0.0)	2(2.0)	2(1.0)	
**Monthly income**				
Less than ₦30,000	90(90.0)	89(89.0)	179(89.5)	0.818
≥₦30,000	10(10.0)	11(11.0)	21(10.5)	

Tables 2 and 3 explains the regression analysis and summary of the model analysis with breastfeeding self-efficacy and sociodemographic characteristics of the respondents. A significant relationship was observed between breastfeeding self-efficacy of the women and age (B = 0.24), marital status (6.38) and occupation (3.07) at X^2 =^ 1.95, p = 0.01.

**Table 2:** Regression analysis.

Model	B	SE	T	Sig	95.0% Confidence Interval for B	
					Lower bound	Upper bound
Age	0.239	4.935	2.580	0.11	0.56	0.422
Marital Status	6.379	3.235	2.083	0.39	0.335	13.142
Occupation	3.067	1.406	2.182	0.31	0.284	5.849

**Table 3:** Summary of model analysis.

Model summary	R	R Squares	Adjusted R square	Standard error of estimate	F	Sig
	0.472	0.223	0.108	5.02845	1.946	0.018

In **Table 4,** 49.0% of the participants were in their second trimesters of pregnancy, 77% were multiparous mothers, 73.5% had registered at the antenatal clinic, and 76.0% gave birth to their previous child through normal spontaneous vaginal delivery.

**Table 4:** Pregnancy history of participants.

Variable	Control N (%)	Intervention N (%)	Total	p-value
**Trimester of pregnancy at baseline**				
No Response	3(3.0)	5(5.0)	8(4.0)	0.333
Second trimester (4–6)	54(54.0)	44(44.0)	98(49.0)	
Third trimester (7–9)	43(43.0)	51(51.0)	94(47.0)	
**Parity**				
Multiparous	79(79.0)	75(75.0)	154(77.0)	0.502
Primiparous	21(21.0)	25(25.0)	46(23.0)	
**Mode of delivery of last pregnancy**				
Spontaneous normal delivery	76(76.0)	76(76.0)	152(76.0)	0.837
Caesarean section	1(1.0)	2(2.0)	3(1.5)	
No response	23(23.0)	22(22.0)	45(22.5)	
**Registered for antenatal**				
Yes	78(78.0)	69(69.0)	147(73.5)	0.149
No	22(22.0)	31(31.0)	53(26.5)	

### Breastfeeding initiation of women by study group

**Fig 1:**
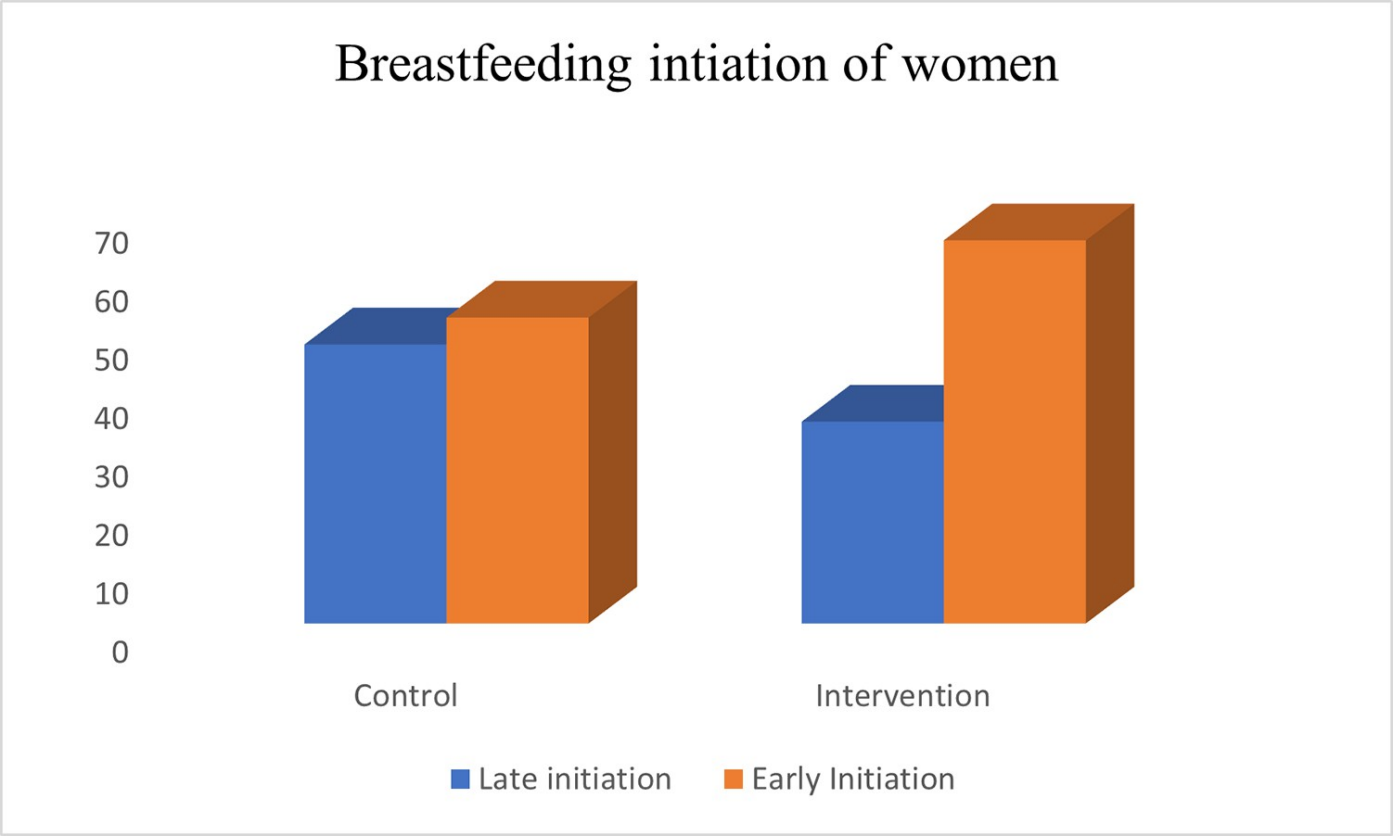
Breastfeeding initiation of women.

In **Fig 1,** 52.3% of the control group initiated breastfeeding within the World Health Organization’s (WHO) recommended period of the first hour of birth, while 65.5% of the experimental group initiated breastfeeding within the WHO recommended standard of one hour, showing a significant difference between the proportion of women who initiated breastfeeding early in the experimental group compared with the control group (p<0.05).

**Fig 2:**
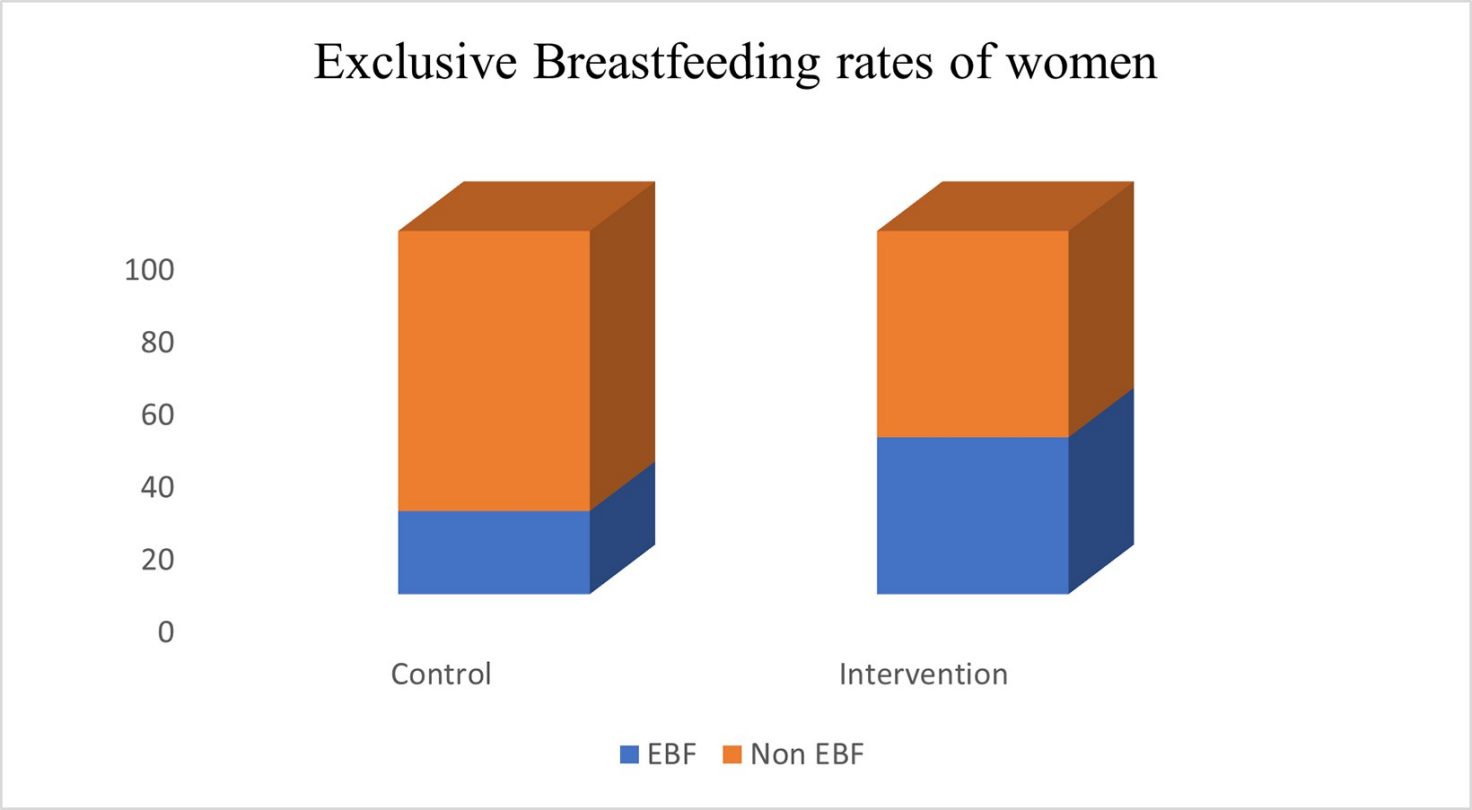
Exclusive breastfeeding practice of women.

In **Fig 2**, 22.9% in the control group practiced EBF, while 43.2% practiced EBF in the experimental group.

**Fig 3:**
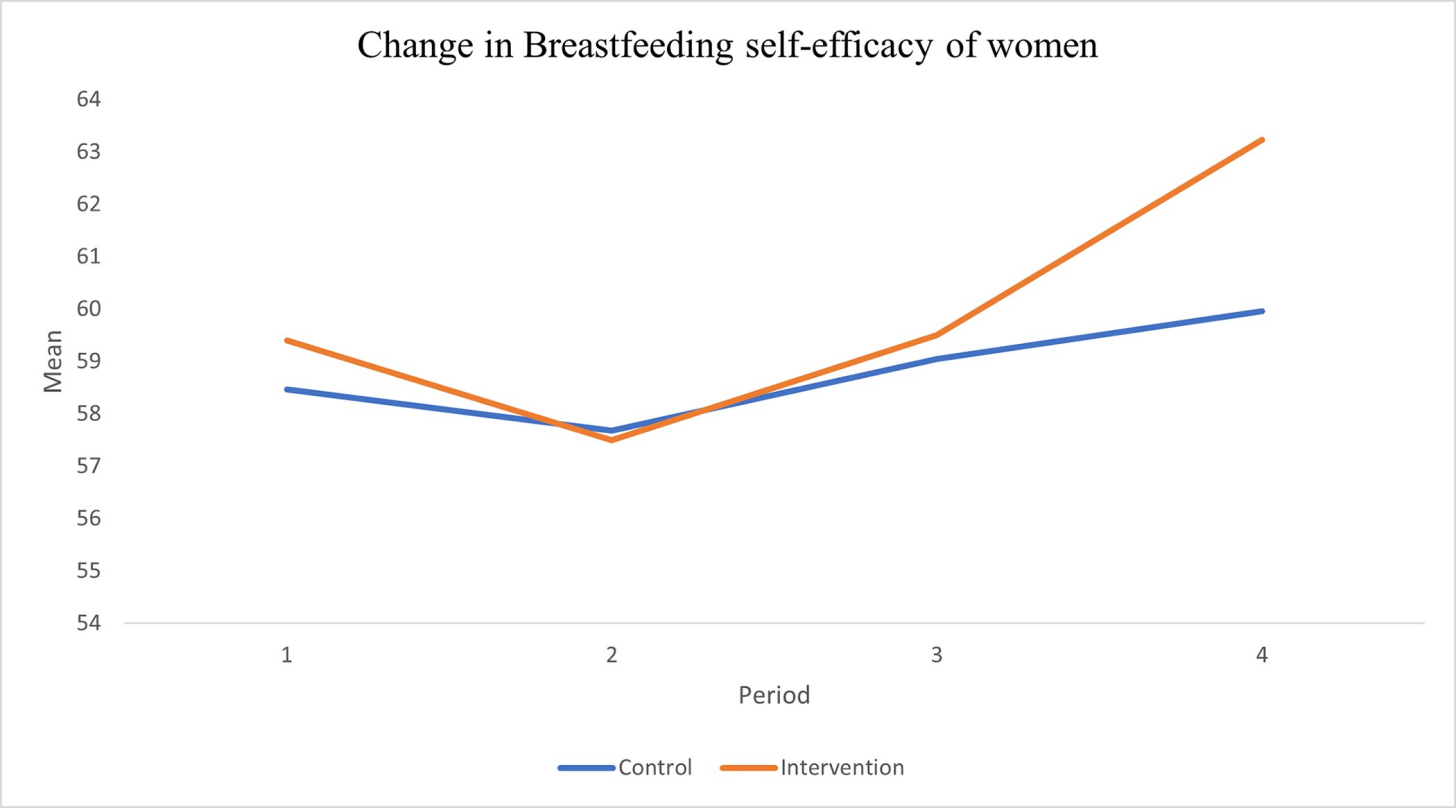
Change in breastfeeding self-efficacy mean score of women.

**Fig 3** explains the change in the breastfeeding self-efficacy (BFSE) mean score of women from baseline (pre-intervention) to endline (one, three and six-months post-intervention). At baseline, the BFSE mean score was 58.47±5.83 and 59.39±7.52 for control and experimental groups respectively. At one month, three months and six months post-intervention, the means scores were 57.68±5.58 and 57.49±5.29; 59.05±5.03 and 59.51±5.47; 59.96±6.36 and 63.23±5.59 for control and experimental groups, respectively. There was an increase in the mean breastfeeding self-efficacy score of women in the experimental group at three months and six months post compared to the control group (p<0.05). Age, marital status, delivery mode, and occupation were identified as predictors of breastfeeding self-efficacy.

## Discussion

The effect of drama-based intervention in improving and BFSE, early breastfeeding initiation and EBF practice of rural mothers from selected communities in two LGAs in Ibadan, Nigeria was investigated. Numerous intervention approaches in different cultural settings and countries have been documented in literature to improve BFSE, breastfeeding initiation and EBF practices of women at 4weeks, 6weeks and 12weeks postpartum. However, for this study, a novel approach which is multidisciplinary in design was employed to determine improvements in BFSE, breastfeeding initiation, EBF practices of mothers at 1week, 4weeks, 12weeks and 24weeks respectively.

The overall mean age of the respondents were 27.0±6.2 and 28.4±6.50 years for experimental and control groups respectively, with higher percentage within the age range of 20-29years. Most of these research participants were married and reside in the selected rural communities. However, this is not surprising since the respondents might have grown up in the area or villages that shared close proximity. The age range of respondents in this study is similar to studies reported in Ethiopia [12], Ogun state Nigeria [13], and Uganda [14]. Also, more than half of the respondents had secondary school education. Moreover, many were either artisans or traders and earned less than thirty thousand naira (₦30,000.00) minimum wage per month. This finding is consistent with the result of studies done in South-east Nigeria [15], and Uganda [14].

About half of the respondents from the two LGAs were recruited in their second trimester of pregnancy owing to the fact that some women might still be experiencing menstrual flow at an early stage of pregnancy. More so, majority of these respondents were young adults. This experience is commonplace with rural women who might not have easy access to health care facilities due to distance. Majority of the respondents were multiparous, registered for antenatal clinic and had spontaneous vaginal delivery. This report is a deviation from the common practice of the rural women registering with traditional birth attendants which can worsen pregnancy and childbirth outcomes, especially when there are complications as reported in studies conducted in Ogun State, Nigeria [13], and Uganda [14].

### Breastfeeding self-efficacy

Evidence from previous studies have identified numerous factors that affect the BFSE of women, some of which are: age, previous breastfeeding experience, mothers’ attitudes, encouragement from family members and friends, nipple pains, nipple shape, stress and anxiety, low birth weight, employment, and mode of delivery [16]. The overall BFSE mean score of those in the intervention group increased significantly from baseline ((59.39±7.52) to six months postpartum (63.23±5.59) as compared with those in the control group (58.47±5.83, and 59.96±6.36); showing the positive effect of drama intervention on the participants. The results from this study also reveal that BFSE mean score at baseline were higher than 46.4 reported in Iran [9], 55.8 in Columbia [4], and 55.5 in Poland [9]. The observed higher values for both intervention and control groups might have been due to the influence of previous knowledge received by participants from healthcare facilities since most of them were multiparous. The observed increase in the BFSE is in line with the results of intervention work by other researchers in Vietnam [5], Hong Kong [6], Iran [17], Brazil [10, 18], Japan [19, 20], Iran [21], Cyprus [22], Taiwan [23], Canada [24] and Iran [25] who used workshop, workbooks, pamphlets, videos, movies, flipcharts, peer educators, and telephone. Also, the relationship between socio-demographic characteristics and BFSE of the participants showed that marital status and mode of delivery had positive significant association on BFSE. This is similar to the findings in Portugese [26], Egypt [27], Turkey [28], Ahzat, Iran [29], Tabriz Iran [30], Khorramabad Iran [31], Denmark [32], River State, Nigeria [33], Osun State Nigeria [34], Abuja, Nigeria [35].

### Breastfeeding initiation and exclusivity

Majority, 65.5% of mothers in the experimental group initiated breastfeeding within the recommended one hour of birth, while 52.3% of those in the control group also initiated breastfeeding within this period. The observed early initiation of breastfeeding is an improvement over the traditional practice of late initiation of breastfeeding and restriction of child feeding with colostrum which is believed to be dirty and unhealthy. The early initiation of breastfeeding among the participants in this study might have been due in part to the level of education of the women at the healthcare centres, and majorly due the effect of drama intervention in the intervention group. The results of the early initiation observed in this study is similar to what was observed in previous studies conducted in Iran [36], Nigeria [37], Turkey [38], Nebraska [39], Jordan [40], Iran [41], India [42], Taiwan [43] and Ethiopia [44, 45] on importance of breastfeeding education or motivation to increase breastfeeding initiation among mothers.

At six months postpartum, 43.2% out of 65.5% who initiated early breastfeeding in the intervention group were able to practice EBF for the period of six months while 22.9% out of 52.3% who initiated EBF in the control group were also able to practice EBF for six months. This finding shows a positive impact of the drama intervention on early breastfeeding initiation of mothers as well as EBF practices which is also consistent with the studies reported in Australia [46], Iran [36] and Brazil [10].

Qualitative findings on the experience of mothers on EBF practices revealed nipple pain, type of employment, insufficient breastmilk, thought of babies having Sunken Fontanel, weight loss, frequent meals, baby being thirsty, and sleepless nights as some of the reasons for early breastfeeding cessation. These result mirrors the findings in Ghana [47], Saudi Arabia [48], Italy [49], Egypt [27], Turkey [28] and Iran [30] on the cultural belief and practices of EBF.

Identified factors that could affect EBF practices of the mothers were age, marital status, education, and occupation. This finding agrees with the result of the study done in Mashhad Iran [21], Cyprus [22], Saudi Arabia [24], Canada [50], Khorramabad Iran [31], and Ahvaz Iran [29] as most women who are married tend to have support from their partners on BFSE, thereby enhancing their practice of EBF.

### Strength and limitations

The strength of this study lies in the adoption of a mixed method for data collection. The qualitative findings identified some cultural and contextual factors that affected the practice of EBF which is similar with previous work in Khorramabad Iran [31], Nigeria [29] and Indonesia [16]. Secondly, assessment of BFES was examined at four-point intervals. We were able to examine the change in BSES at each interval and also the prevalence of EBF at six months postpartum. In addition, the monitoring of the delivery time and day of the mothers for breastfeeding initiation data collection was very useful. Regarding the limitation, a total 21.5% out the initial sample size was lost to lost to follow up at one month, three and six months post data collection. Therefore, further research is needed to consider all of these cultural and contextual factors to improve breastfeeding initiation, exclusivity and breastfeeding self-efficacy of mothers.

### Conclusion

There was an increase in the BFSE, breastfeeding initiation, and EBF of women in the experimental group compared to those in the control group from baseline to six months postpartum. The drama intervention was therefore effective in the increase of BFSE to achieve the WHO recommended EBF rate. The practice of EBF and mothers’ BFSE was also found to be influenced by predictors such maternal age, marital status, occupation, and education. To increase the exclusivity rate, policies should also be developed on the use of educational entertainment by healthcare professionals, such as theater.
